# Effect of Rare-Earth Co-Doping on the Trap Level Concentrations in Silica Glasses: Experimental and Theoretical Study of the Light Emission Under X-Rays for Dosimetry Applications

**DOI:** 10.3390/s25103005

**Published:** 2025-05-09

**Authors:** Ismail Zghari, Hicham El Hamzaoui, Adriana Morana, Youcef Ouerdane, Bruno Capoen, Sarah Garzandat, Sylvain Girard, Aziz Boukenter, Franck Mady, Mourad Benabdesselam, Gilles Mélin, Mohamed Bouazaoui

**Affiliations:** 1 Univ. Lille, CNRS, UMR 8523-PhLAM-Physique des Lasers Atomes et Molécules, F-59000 Lille, France; hicham.el-hamzaoui@univ-lille.fr (H.E.H.); bruno.capoen@univ-lille.fr (B.C.); sarah.garzandat@univ-lille.fr (S.G.); 2 Université Jean Monnet Saint-Etienne, CNRS, Institut d’Optique Graduate School, Laboratoire Hubert Curien UMR 5516, F-42023 Saint-Etienne, France; adriana.morana@univ-st-etienne.fr (A.M.); ouerdane@univ-st-etienne.fr (Y.O.); sylvain.girard@univ-st-etienne.fr (S.G.); aziz.boukenter@univ-st-etienne.fr (A.B.); 3 Institut Universitaire de France (IUF) Ministère de l’Enseignement Supérieur et de la Recherche, 1 Rue Descartes, F-75005 Paris, France; 4 Université Côte d’Azur, CNRS, Institut de Physique de Nice—INPHYNI UMR 7010, F-06108 Nice Cedex 2, France; franck.mady@unice.fr (F.M.); mourad.benabdesselam@unice.fr (M.B.); 5 Exail, Rue Paul Sabatier, F-22300 Lannion, France; gilles.melin@exail.com

**Keywords:** fiber sensors, silica glass, X-ray, rare-earth, radioluminescence, thermoluminescence

## Abstract

In this paper, an experimental and theoretical study was undertaken to assess the impact of rare-earth co-doping of silica glasses on the light emission under X-rays. To this aim, radioluminescence (RL), phosphorescence (PP), and thermoluminescence (TL) signals of Ce^3+^/Gd^3+^ co-doped silica glasses have been successively measured and combined at different dose rates and irradiation temperatures. The RL response of the weakly co-doped sample was found to be temperature-independent between 273 K and 353 K. This result suggests that, based on this RL response, it is possible to design ionizing radiation sensors independent of the irradiation temperature in the corresponding range. Moreover, a model that considers the electron–hole pair generation, the charge carrier trapping–detrapping, and the electron–hole recombination in the localized and delocalized bands has been developed to reproduce these optical signals. The theoretical model also explains the temperature independence of the RL response between 273 K and 353 K for the weakly co-doped sample and, therefore, the operating principle of an X-ray sensor independent of the irradiation temperature.

## 1. Introduction

Silica-based optical fiber glasses have, for decades, aroused interest among researchers in the field of ionizing radiation detection due to their multiple advantages compared to other dosimetry systems [1,2,3,4,5,6,7,8]. These optical fibers are almost tissue equivalent. They can be distinguished by their higher radiation-hardening in comparison to organic materials [9,10,11]. In addition, they are insensitive to electromagnetic interferences and offer high spatial resolution thanks to their small sensitive volume. Their cylindrical symmetry makes their response independent of the radiation beam angle around their fiber axes. Furthermore, silica-based fibers exhibit a linear response over a wide range of dose/dose rates and enable real-time measurements [12,13,14,15,16]. Fibered systems also offer the possibility of performing remote measurements without exposing the fragile electronic acquisition instruments (photodetectors) to harsh radiation environments. Dosimetry based on optical fibers has demonstrated its potential in many fields of applications, notably in radiotherapy for machine quality control and in vivo dosimetry during patient treatments [17,18,19]. It also finds essential applications in the space sector [9], particularly during missions to quantify the dose absorbed by astronauts. Furthermore, these silica-based optical fiber systems are highly recommended for the nuclear field, whether in nuclear facilities or in nuclear waste storage repositories [20,21].

The main phenomena involved in optical dosimetry are radio-induced emission (RIE), thermoluminescence (TL), and radiation-induced attenuation (RIA) in fibers [22]. RIE includes radioluminescence (RL) and Cerenkov emissions but the latter is produced only under high energy beams. This study concerns the RL, phosphorescence (PP), and TL processes acquired from doped silica rods spliced to optical fibers. It is well known that these processes are strongly dependent on different characteristics of the scintillating material, like the recombination centers, the amorphous or crystalline nature of the matrix, and the presence of defects as a function of composition. In this paper, we focus on the potential relationship between the rare-earth doping of silica glass and the defect concentrations, as well as their impact on RL and TL behaviors. It is known that rare-earth elements are widely used as luminescent centers in glasses and particularly as scintillators [23].

Hence, this study aims to sequentially measure RL, PP, and TL signals of Ce^3+^/Gd^3+^ co-doped silica glasses, at different dose rates and irradiation temperatures by using the same experimental setup. The choice of Ce^3+^ and Gd^3+^ co-doping is motivated by the achievement of dosimeters sensitive to different ionizing radiations (X-rays, protons…). In particular, Gd^3+^ ions are convenient for proton dosimetry [5]. In effect, the protons could generate neutrons inside the doped glass. According to the literature [24], Gd presents the highest neutron absorption cross sections compared to Ce, leading to an RL signal. The corresponding absorption cross sections are of about 242,000 and 0.66 barn for Gd^157^ and Ce^140^, respectively. A model that takes into account the electron–hole pair generation, the charge carrier trapping–detrapping, and the electron–hole recombination in the localized and delocalized bands has been developed to reproduce these optical signals. Such a simulation can give access to trap-level concentration for each investigated sample and allows comparison between their optical properties in various experimental conditions (dose rate, temperature).

## 2. Materials and Methods

### 2.1. Studied Samples

Ce^3+^/Gd^3+^ co-doped samples were fabricated using the sol-gel method at FiberTech Lille platform of the University of Lille, as described elsewhere [1]. Briefly, porous silica xerogels were synthetized from a tetraethylorthosilicate precursor. These xerogels were stabilized at 1000 °C and then soaked in alcoholic solutions containing cerium and gadolinium salts. Afterward, the samples were removed from the doping solutions and dried at a temperature of 50 °C for one day to eliminate solvents and retain the doping ions. The doped matrices were then sintered under helium flux at 1300 °C for 2 h. Finally, the co-doped vitreous rods were drawn at a temperature of about 2000 °C.

The Ce/Gd-co-doped materials studied here were the same as in [15]. The sample labels and the corresponding concentrations of RE elements, determined using electron probe microanalysis (EPMA), are summarized in Table 1. For dosimetry measurements, the experimental setup consists of a 1 cm-long piece of the co-doped cane with a 220 µm diameter welded to an optical fiber with a length of 5 m and with the same diameter as the co-doped rods. This fiber was employed to transport the optical signal.

### 2.2. TL and Initial Rise Experimental Setup

Thermally stimulated luminescence (TL) measurements were achieved on Ce/Gd-doped silica samples from RT to 600 K, with a linear heating rate of 1 K/s. The laboratory-made TL system makes use of a photomultiplier tube (PMT), with spectral sensitivity from 250 nm to 650 nm. The sample was irradiated at INPHYNI to a dose of about 722 Gy using an XRG3D X-ray tube (INEL, Artenay, France) with a copper anode operated at 30 kV. The irradiation was calibrated by a PTW 23342 ionization chamber (PTW, Freiburg, Germany) and the delay between the irradiation and the TL reading was 2 min. The initial rise method first involves irradiating the sample, followed by heating it in order to obtain a complete thermogram, continuing until the temperature reaches a level that empties all the traps. This step notably allows for the determination of the maximum intensity ($I_{m}$) at temperature $T_{m}$. Next, the sample is subjected to a second identical irradiation, followed by gradual heating until the measured intensity reaches 10% of $I_{m}$, corresponding to a temperature $T_{stop1}$. After this, the sample is cooled to room temperature. A second heating is then applied up to a temperature $T_{stop2}$, where the intensity $I_{2}$ again reaches 10% of $I_{m}$, followed once more by cooling to room temperature. This methodology is repeated until all traps are emptied. A detailed description of the calculation steps is provided in [25].

### 2.3. RL, PP and FTSL Measurements

The dosimetry measurements consist of collecting the light emission from the glassy rods during and after X-ray exposure. The X-ray beam was provided by the LABHX facility of Laboratoire Hubert Curien, functioning at 100 kV and producing photons of ~40 keV average energy. The doped sample was positioned in a calibrated part of the irradiator. Under X-rays, the RL optical signal was then guided via the transport fiber to a photomultiplier module (PMT, H9305-13, Hamamatsu, Japan). The acquisition was assured through a photon-counting unit. The dose rate at the various positions had been evaluated with an ionization chamber. Dose rates and doses have been assessed considering the ratio between mass attenuation coefficients μ/ρ of water and silica, where μ and ρ are the photon attenuation coefficient and the medium density, respectively. Doses are consequently expressed in Gy (silica).

RL spectra were recorded at different temperatures after replacing the PMT detector with a UV/VIS mini-spectrometer (Ocean Insight QE PRO, Orlando, FL, USA). Before analysis, the dark signal, due to the device, was subtracted from the RL signal. The temperature of the sample was changed by pasting the transport fiber to a temperature-controlled plate associated with a type K thermocouple, and for each temperature, before irradiation, a thermalization time of 10 min was adopted.

Our measurement conditions exclude the Cerenkov effect, thanks to the use of X-ray photons having an average energy of 40 keV. Additionally, we have taken precautions to reduce any eventual noise signal coming from the ionization of the transport fiber by covering it with lead plates.

The measurements were carried out over a temperature range of 153–353 K. After having cautiously placed the sample in the calibrated location on the temperature-controlled plate, the experimental protocol proceeded as follows: at each irradiation temperature, the sample was irradiated for 5 min at a specific dose rate (RL), and then, data acquisition was continued for 3 min without X-ray irradiation, enabling detection of the PP signal. Then, the TL measurement was recorded by heating the sample from the irradiation temperature ($T_{irr}$) to 353 K, with a heating rate of 5 K/min. The detection of TL in doped glasses via an optical fiber is new; this is why we have named it fibered thermo-stimulated luminescence (FTSL). Indeed, the novelty of this method is based on the use of an optical fiber to remotely collect the TL emission from the luminescent sample. To our knowledge up to now, the TL of optical fibers is mainly obtained on grounded optical fiber using standard TL equipment. The dose rates used in this study covered a range from 50 to 1000 mGy/s.

## 3. Experimental Results

### 3.1. RL Spectra

**Figure 1:**
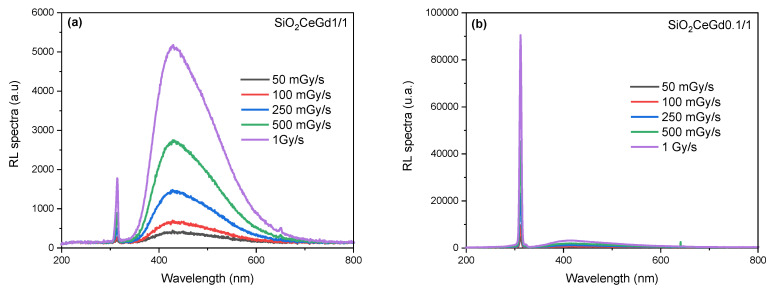
RL spectra obtained under different X-ray dose rates ranging from 50 to 1000 mGy/s at irradiation temperature of 153 K for (**a**) SiO_2_CeGd1/1 and (**b**) SiO_2_CeGd0.1/1.

Figure 1 displays the RL spectra of both co-doped samples obtained at a temperature of 153 K under various X-ray dose rates ranging from 50 to 1000 mGy/s. They reveal two distinct emissions: a broad emission band extending approximately from 350 nm to 650 nm, peaking at around 460 nm, and a narrower one centered around 314 nm. The first emission is attributed to the allowed 5d → 4f optical transition of Ce^3+^ ions [1,13,26,27,28], and the second one is assigned to the radiative transition between the ^6^P_7/2_ and ^8^S_7/2_ levels of Gd^3+^ [29,30,31]. Furthermore, it is notable that in Figure 1a, the emission of Ce^3+^ in the SiO_2_CeGd1/1 sample was more pronounced compared to the one of Gd^3+^, while for SiO_2_CeGd0.1/1, the opposite is observed (Figure 1b). This reflects the effect of Ce concentration in each sample. Accordingly, these results demonstrate that the Ce^3+^ and Gd^3+^ ions constitute the recombination centers at the origin of the optical signal. Several works show that in an oxide, cerium and gadolinium can exist in the tetravalent state [1,32,33]. This is why we will attribute both Ce^3+^ and Gd^3+^ to hole centers in the model of Section 4.

### 3.2. TL and Initial Rise

**Figure 2:**
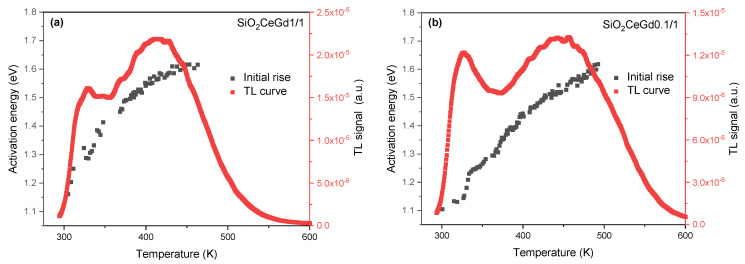
TL measurements performed on the SiO_2_CeGd1/1 (**a**) and SiO_2_CeGd0.1/1 (**b**) samples, in the temperature range from RT to 600 K along with the calculated activation energies against the stop temperature using the partial-cleaning initial rise method.

Figure 2 depicts TL measurements conducted on SiO_2_CeGd1/1 and SiO_2_CeGd0.1/1 in the temperature range from RT to 600 K, along with the distribution of trap levels, which is estimated using the partial-cleaning initial rise method. The thermoluminescence curves of both samples reveal two main peaks at around 350 and 460 K. The peak below 350 K is always present in the TL curve of silica-based materials and probably associated with Si-ODC defects [34,35]. The second peak, at about 450 K, is induced by the doping with RE ions [34]. In the case of SiO_2_CeGd1/1, the maximum intensity of the first peak is lower than that of the second peak. Conversely, for the SiO_2_CeGd0.1/1 sample, the maximum intensities of the two peaks are almost equal. Consequently, this leads us to conclude that the matrix is sensitive to the concentration of dopants. Furthermore, this optical signal arises from the detrapping of charge carriers in both samples, demonstrating an almost identical continuous distribution of trap levels between 1.1 and 1.6 eV. The continuous character of this distribution results from the amorphous nature of the SiO_2_ matrix. This distribution was estimated using the initial rise method from room temperature to 500 K. These energy values will be considered in our model to simulate the sample emission induced by exposure to X-rays (RL, PP, and FTSL). For trap levels corresponding to the lowest temperatures, the energy values are taken from the literature [36].

### 3.3. RL, PP, and FTSL Signal

**Figure 3:**
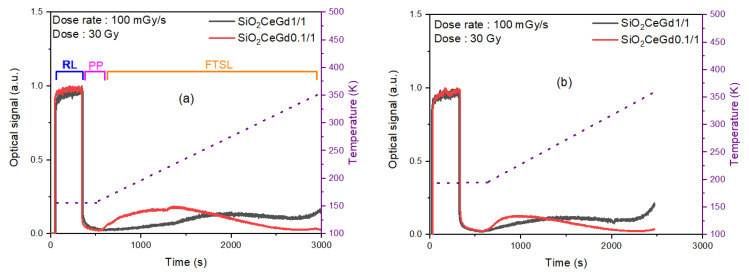
Optical signal response of both Ce/Gd-co-doped silica samples for an X-ray dose rate of 100 mGy/s (accumulated dose of 30 Gy) and at two different irradiation temperatures ($T_{irr}$) of 153 K and 193 K. Each RL curve is followed by phosphorescence and then by FTSL from the $T_{irr}$ to 353 K with a heating rate of 5 K/min, starting from (**a**) 153 K and (**b**) 193 K.

Figure 3 illustrates the time-dependent dynamics of the optical signal obtained by exposing SiO_2_CeGd1/1 and SiO_2_CeGd0.1/1 samples to an X-ray dose rate of 100 mGy/s for 300 s (accumulated dose of 30 Gy) at two different irradiation temperatures ($T_{irr}$) of 153 K and 193 K. Each RL curve (in the blue zone) is followed when the irradiation is stopped, by a phosphorescence (PP) signal for 180 s (in the pink zone) at the same temperature $T_{irr}$ and then, when increasing the temperature up to 353 K with a 5 K/min heating rate, by an FTSL signal (in the orange zone). The discussion of the time evolution of the RL response during and after the end of irradiation is detailed in references [37,38]. Additionally, it is important to note that the FTSL response differs between the two samples. Indeed, after PP, the FTSL signal of SiO_2_CeGd1/1 features a slow increase to reach a maximum at around 2000 s for $T_{irr}$ = 153 K (Figure 3a—black curve) and 1500 s for $T_{irr}$ = 193 K (Figure 3b—black curve). The temperature of the FTSL maximum is roughly the same, corresponding to 275 K, for both irradiation temperatures ($T_{irr}$ = 153 K and $T_{irr}$ = 193 K). In Figure 3a—black curve—we observe a very weak signal between 600 and 1000 s, corresponding to a 153–190 K interval. In contrast, in the case of SiO_2_CeGd0.1/1, the FTSL signal initiates a rapid increase with a maximum around 1300 s for $T_{irr}$ = 153 K (Figure 3a—red curve) and 1000 s for $T_{irr}$ = 193 K (Figure 3b—red curve), corresponding to 210 K. In Figure 3a—red curve—the maximum is followed by a gradual decrease to reach a very weak signal from about 2500 s (311 K) up to 353 K. This difference in FTSL responses reflects distinct trap level concentrations in the corresponding samples, as seen above in TL measurements. To assess the relative concentrations of traps, we have developed a model to simulate the entire luminescence signal, including RL, PP, and FTSL.

**Figure 4:**
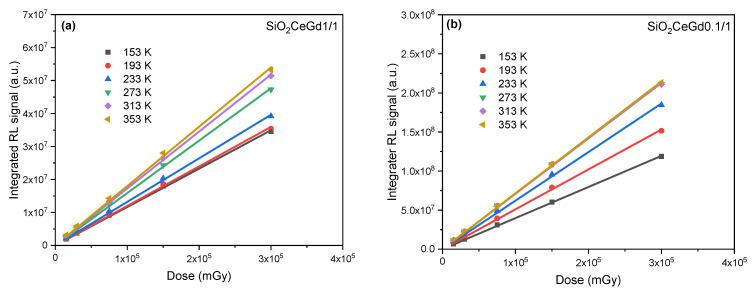
Integrated RL response versus X-ray dose at different irradiation temperatures ranging from 153 K to 353 K of (**a**) SiO_2_-CeGd1/1 and (**b**) SiO_2_-CeGd0.1/1.

In Figure 4, we have plotted the evolution of SiO_2_CeGd1/1 and SiO_2_CeGd0.1/1 integrated RL signals alone (the blue zone in Figure 3) as a function of X-ray dose for different irradiation temperatures. For both samples, the RL signal exhibits a linear behavior which increases against the dose for all these temperatures ranging from 153 to 353 K. Furthermore, it can be noted that the RL response of SiO_2_CeGd0.1/1 is temperature-independent between 273 K and 353 K.

## 4. Discussion

The mathematical model that will be used here to simulate our experimental data is an extension of the one previously presented in [37,38].

It describes the electron–hole pair generation, the carrier charge trapping–detrapping, and the electron–hole recombination result in the following set of coupled differential equations:

(1) $$\frac{dn_{c}}{dt}=X-\sum_{i=1}^{k}n_{c}\left(N_{i}-n_{i}\right){A_{e}}_{i}+\sum_{i=1}^{k}n_{i}s_{i}\exp\left(\frac{-E_{i}}{k_{B}T}\right)\;-{\alpha_{1}n}_{c}A_{r1}m_{1}-{\alpha_{2}n}_{c}A_{r2}m_{2}$$

(2) $$\frac{dn_{i}}{dt}=n_{c}\left(N_{i}-n_{i}\right){A_{e}}_{i}-n_{i}s_{i}\exp\left(\frac{-E_{i}}{k_{B}T}\right)\;(i=1,\;\ldots .,\;k)\;$$

(3) $$\frac{dm_{v}}{dt}=X-\sum_{j=1}^{2}m_{v}\left(M_{j}-m_{j}\right)A_{hj}$$

(4) $$\frac{dm_{j}}{dt}=m_{v}\left(M_{j}-m_{j}\right)A_{hj}-\alpha_{j}n_{c}A_{rj}m_{j}\;(j=1,\;2)$$

(5) $$I=n_{c}\left({\alpha_{1}m}_{1}A_{r1}+{\alpha_{2}m}_{2}A_{r2}\right)$$

All the parameters used in this model are reported and defined in Table 2. The major difference with our previous model [37,38] is related to the necessity of considering more than one recombination center, since the material is co-doped with two optically active centers (Ce^3+^ and Gd^3+^). This results in the addition of one new differential equation governing the carrier recombination on this second center in line (4), associated with the transition probabilities $A_{hj}$ and $A_{rj}$. The emission ratios $\alpha_{j}$ are deduced from the RL spectra shown in Figure 1, and each of these parameters represents the relative contribution of each recombination center to the total signal.

The above equations describe the time variation of (1) the electron concentration in the conduction band (CB), (2) the electron concentration in the *i*th trap, (3) the hole concentration in the valence band (VB), (4) the hole concentration in the *j*th RC, and (5) the emission intensity. It should be stressed that non-radiative recombination is not taken into account. About solving the differential equations, determining parameter values, as well as detailing the simulation steps associated with each mechanism (RL, PP, and FTSL), a comprehensive explanation can be found in our previous work [39].

In this study, 50 trap levels were used to simulate the experimental emission curves using the parameter values presented in Table 2. This number of traps represents the minimum required for an accurate modeling of the experimental results. It strikes a balance between an adequate energy resolution (0.015 eV) and a simplified management of the calculation program’s parameters. It should be noted that, for each sample, these values remain unchanged for all investigated dose rates and temperatures.

**Figure 5:**
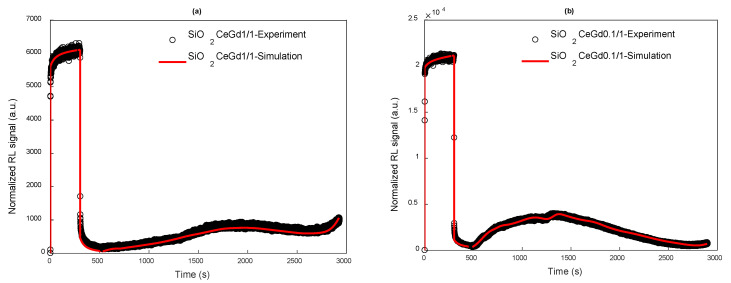
Measured and simulated RL + PP + FTSL signals with 50 traps ranging from 0.43 to 1.5 eV under CB and two RC for a dose rate of 100 mGy/s (accumulated dose 30 Gy) at temperature of 153 K for (**a**) SiO_2_CeGd1/1 and (**b**) SiO_2_CeGd0.1/1.

Figure 5 presents the simulation results of the emission signal of the two samples, SiO_2_CeGd1/1 and SiO_2_CeGd0.1/1, as a function of time (RL followed by PP and FTSL) from the irradiation temperature up to 353 K. The simulation curves were multiplied by a factor (F) to fit the experimental curves because the theoretical intensity of the optical signal corresponds to the number of radiative electron–hole recombination events per unit volume and time, while the experimental intensity, measured by the opto-electric conversion of the PMT, is proportional to the number of photons per unit of time. The numerical simulations satisfactorily reproduce the experimental data both during irradiation (RL) and after stopping the irradiation (PP + FTSL). This agreement between the experimental results and the simulations was obtained for all other temperatures and for all doses with the same multiplying factor F. Even more remarkable is that this good agreement was achieved using a single set of parameter values for a given sample.

**Figure 6:**
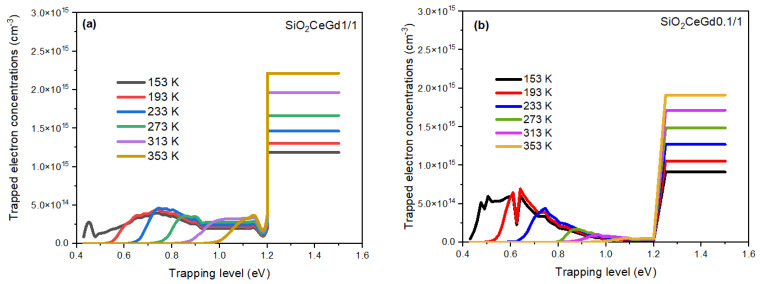
Trapped electron concentration as a function of trap energy level resulting from the simulation at different irradiation temperatures ($T_{irr}$) and at the end of irradiation with a dose rate of 100 mGy/s (accumulated dose of 30 Gy) for (**a**) SiO_2_CeGd1/1 and (**b**) SiO_2_CeGd0.1/1.

Figure 6 illustrates the variation of the trapped electron concentrations as a function of the trap energy levels for the SiO_2_CeGd1/1 and SiO_2_CeGd0.1/1 samples at different irradiation temperatures. Each curve represents the concentration of trapped electrons ($n_{i}$) at the end of irradiation (at 300 s), calculated according to Equation (2), for a dose of 30 Gy (100 mGy/s for 300 s).

As the temperature increases, the concentration of electrons trapped in the shallower energy levels decreases. This decrease results in an increase in free electrons available for recombination during the irradiation step (RL). Consequently, this results in an increase in RL signal as a function of the irradiation temperature ($T_{irr}$).

Additionally, it should be noted that the trapped electron concentration curves vary significantly between the two samples. In the case of SiO_2_CeGd1/1, the concentration shows a gradual increase before reaching maxima around 0.75 eV, followed by a progressive decrease. Furthermore, the concentration of trapped electrons remains significant up to 1.2 eV. On the other hand, SiO_2_CeGd0.1/1 shows trapped electron concentration curves starting with a rapid rise, with maxima around 0.5 eV, then followed by a rapid decrease to reach very low concentrations between 0.7 and 1.1 eV. Thus, the disparity in dopant concentration impacts the density of trap states ($N_{i}$), clearly indicating the sensitivity of the silica matrix to the dopant ion concentration. For better visualization of the trap states ($N_{i}$) presented in Table 2, they have been represented versus energy in Figure S1.

**Figure 7:**
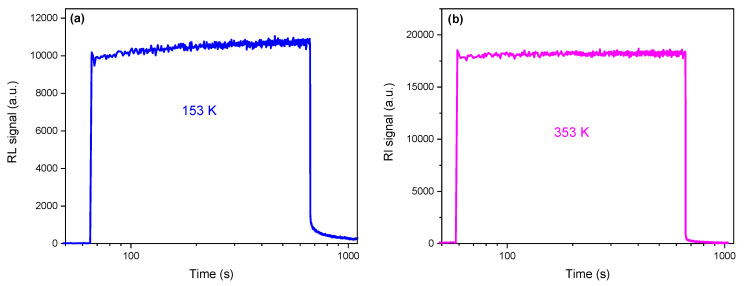
Time evolution of the RL signal measured on SiO_2_CeGd0.1/1 at a dose rate of 50 mGy/s at a temperature of (**a**) 153 K and (**b**) 353 K.

Figure 7 shows the temporal evolution of the RL signal for the SiO_2_CeGd0.1/1 sample at a dose rate of 50 mGy/s and at temperatures of 153 K and 353 K. It is clearly observable that the RL signal shapes between the two responses are different. The RL response at 153 K continuously increases for a few minutes before reaching the pseudo-plateau. Moreover, after the irradiation is stopped, the optical signal (PP) remains significant and does not reach zero, even after 180 s. On the other hand, at 353 K, the RL signal reaches a plateau very quickly. Furthermore, the PP signal is weak and reaches zero after about 120 s. These different behaviors and dynamics of the RL and PP responses at different irradiation temperatures for the same sample can be explained by the trapping/detrapping kinetics of the metastable levels, as reported in [37,38,39]. Indeed, as shown in Table 2, the metastable trap levels involved at 153 K are $E_{1},E_{2},E_{3},E_{4},$ and $E_{5}$, (in blue) corresponding to high concentrations $N_{1},N_{2},\;N_{3},N_{4},$ and $N_{5}$. However, the metastable traps involved at 353 K are $E_{38},E_{39},E_{40},E_{41},$ and $E_{42}$, (in red) corresponding to low concentrations $N_{38},N_{39},N_{40},N_{41},$ and $N_{42}$. This leads to the conclusion that the lower the concentration of metastable traps, the faster the RL response reaches the plateau.

## 5. Conclusions

In this study, RL, PP, and FTSL signals have been collected from Ce/Gd co-doped silica rods using an optical fiber at different irradiation temperatures. These two co-doped samples differ only by the amount of rare-earth ion doping. Experimental results showed that for both samples and at different irradiation temperatures ($T_{irr}$), the RL response behaves linearly as a function of the dose rate. However, the RL response of the weakly co-doped sample SiO_2_CeGd0.1/1 was found to be temperature-independent between 273 K and 353 K. To simulate these experimental results, a theoretical model has been developed using 50 trap levels and two recombination centers (Ce^3+^ and Gd^3+^). The theoretical analysis showed that the RL response is essentially governed by the trap level distribution in the silica bandgap, which is itself strongly dependent on the rare-earth ion concentrations within the silica matrix. Indeed, it was found that the weakly co-doped sample SiO_2_CeGd0.1/1 presents a low density of traps, whose energies lay between 0.88 and 1.11 eV, explaining the temperature independence of the RL response between 273 K and 353 K.

In the context of fibered dosimeter systems, particularly in the field of flash therapy where irradiation pulses are short and repetition rate is high, several key properties are essential to ensure accurate and rapid dose measurement. A rapid rise time of the RL signal to reach a constant steady-state is necessary to obtain a stable and reliable readout throughout the irradiation period. Moreover, negligible PP is important to avoid any light persistence effects that could distort subsequent measurements. To meet these requirements, material engineering should focus on reducing the concentration of metastable traps corresponding to the specific temperature operating conditions for each application (flash therapy, etc.).

## Figures and Tables

**Table 1:** Sample labels and the corresponding concentrations of RE elements, determined by EPMA.

Symbol	[Ce] (wt.%)	[Gd] (wt.%)
SiO_2_-CeGd1/1	0.07	0.08
SiO_2_-CeGd0.1/1	0.008	0.13

**Table 2:** Parameter values used in the simulations of optical signal emission.

Parameter	Status	Description	Value for *k* = 50 (Number of Electron Traps) SiO_2_CeGd1/1	Value for *k* = 50 (Number of Electron Traps) SiO_2_CeGd0.1/1
$E_{i}\;(eV)$	Fixed	Trapping level	0.43, 0.445, 0.46, 0.475, 0.49, 0.505, 0.52, 0.535, 0.55, 0.565, 0.58, 0.595, 0.61, 0.625, 0.64, 0.655, 0.67, 0.685, 0.7, 0.715, 0.73, 0.745, 0.76, 0.775, 0.79, 0.805, 0.82, 0.835, 0.85, 0.865, 0.88, 0.895, 0.91, 0.925, 0.94, 0.955, 0.97, 0.985, 1, 1.08, 1.095, 1.11, 1.125, 1.15, 1.2, 1.25, 1.3, 1.35, 1.4, 1.5	0.43, 0.445, 0.46, 0.475, 0.49, 0.505, 0.52, 0.535, 0.55, 0.565, 0.58, 0.595, 0.61, 0.625, 0.64, 0.655, 0.67, 0.685, 0.7, 0.715, 0.73, 0.745, 0.76, 0.775, 0.79, 0.805, 0.82, 0.835, 0.85, 0.865, 0.88, 0.895, 0.91, 0.925, 0.94, 0.955, 0.97, 0.985, 1, 1.08, 1.095, 1.11, 1.125, 1.15, 1.2, 1.25, 1.3, 1.35, 1.4, 1.5
$N_{i\;}(cm^{-3})$	Free	Concentrations of electrons traps	4.5 × 10^19^, 4.5 × 10^19^, 2 × 10^19^, 6 × 10^18^, 6.5 × 10^18^, 7 × 10^18^, 7.5 × 10^18^, 8.5 × 10^18^, 9.5 × 10^18^, 1 × 10^19^, 1.1 × 10^19^, 1.2 × 10^19^, 1.3 × 10^19^, 1.4 × 10^19^, 1.6×10^19^, 1.7 × 10^19^, 1.7 × 10^19^, 1.8 × 10^19^, 1.8 × 10^19^, 1.9 × 10^19^, 2 × 10^19^, 2 × 10^19^, 1.9 × 10^19^, 1.9 × 10^19^, 1.8 × 10^19^, 1.8 × 10^19^, 1.7 × 10^19^, 1.6 × 10^19^, 1.5 × 10^19^, 1.4 × 10^19^, 1.3 × 10^19^, 1.3 × 10^19^, 1.2 × 10^19^, 1 × 10^19^, 1 × 10^19^, 1 × 10^19^, 1 × 10^19^, 1 × 10^19^, 1 × 10^19^, 1 × 10^19^, 1 × 10^19^, 1 × 10^19^, 1 × 10^19^, 1 × 10^19^, 1 × 10^19^, 6 × 10^19^, 6 × 10^19^, 6 × 10^19^, 6 × 10^19^, 6 × 10^19^	4 × 10^19^, 4 × 10^19^, 4 × 10^19^, 5 × 10^19^, 4 × 10^19^; 5 × 10^19^, 4 × 10^19^, 5.5 × 10^19^, 4.5 × 10^19^, 4.5 × 10^19^, 4.5 × 10^19^, 4.5 × 10^19^, 4.5 × 10^19^, 2 × 10^19^, 4.5 × 10^19^, 4 × 10^19^, 3.5 × 10^19^, 3.2 × 10^19^, 3 × 10^19^, 2.5 × 10^19^, 2.4 × 10^19^, 2.3 × 10^19^, 2.1 × 10^19^, 1.9 × 10^19^, 1.65 × 10^19^, 1.45 × 10^19^, 1.3 × 10^19^, 1.2 × 10^19^, 1.1 × 10^19^, 9.5 × 10^18^, 8.5 × 10^18^, 7.5 × 10^18^, 6.5 × 10^18^, 5.5 × 10^18^; 5 × 10^18^, 4.5 × 10^18^, 4 × 10^18^, 3.5 × 10^18^, 3 × 10^18^, 2.5 × 10^18^, 2 × 10^18^, 1.5 × 10^18^, 1.5 × 10^19^, 1.5 × 10^19^, 1.5 × 10^19^, 6 × 10^19^, 6 × 10^19^, 6 × 10^19^, 6 × 10^19^, 6 × 10^19^
$s_{i}\;(s^{-1})$	Fixed	Frequency factors	5 × 10^12^	5 × 10^12^
$\frac{A_{ei}}{A_{r}}$	Free	Ratio between recombination and trapping coefficients	~10^−3^	~10^−3^
$X\;(cm^{-3}.s^{-1})$	Fixed	Rate of electron–hole pairs production	100 mGy(SiO_2_)/s → 8.44 × 10^13^	100 mGy(SiO_2_)/s → 8.44 × 10^13^
$M\;(cm^{-3})$	Fixed	Concentration of radiative hole center	Ce^3+^: 6.62 × 10^18^ Gd^3+^: 6.74 × 10^18^	Ce^3+^: 7.5 × 10^17^ Gd^3+^: 1 × 10^19^
$k_{B}$	Fixed	Boltzmann constant	8.617330 × 10^−5^ eV. K^−1^	8.617330 × 10^−5^ eV. K^−1^
$t_{i}\;(s)$	Fixed	Irradiation time	300	300
$t_{f}\;(s)$	Fixed	Afterglow time	180	180
$n_{i}$ and $n_{c}\;(cm^{-3})$	Free	Density of electrons in $N_{i}$ and CB, respectively	Evolves over time	Evolves over time
m and $m_{v}\;(cm^{-3})$	Free	Density of holes in *RC* and VB, respectively	Evolves over time	Evolves over time
$\alpha_{j}$	Fixed	Emission ratio	$\alpha_{1}=0.982$; $\alpha_{2}=0.018$	$\alpha_{1}=0.554$; $\alpha_{2}=0.446$

## Data Availability

The data that support the findings of this study are available from the corresponding author upon reasonable request.

## Supplementary Materials

The following supporting information can be downloaded at: https://www.mdpi.com/article/10.3390/s25103005/s1, Figure S1: Concentration of trap states (Ni) taken from Table 2, versus energy.
