# Supplementary material for: Effect of Rare-Earth Co-Doping on the Trap Level Concentrations in Silica Glasses: Experimental and Theoretical Study of the Light Emission Under X-Rays for Dosimetry Applications
Source: Sensors (Basel). 2025 May 9;25(10):3005. doi: 10.3390/s25103005 (PMC12115133; doi:10.3390/s25103005)
Supplement: Supplementary file 1 [file sensors-25-03005-s001.zip › sensors-3596101-supplementary.pdf]

Effect of Rare-Earth Co-Doping on the Trap Level Concentrations in Silica Glasses: Experimental and Theoretical Study of the Light Emission Under X-Rays for Dosimetry Applications

Ismail Zghari [1,*], Hicham El Hamzaoui [1], Adriana Morana [2], Youcef Ouerdane [2], Bruno Capoen [1], Sarah Garzandat [1], Sylvain Girard [2,3], Aziz Boukenter [2], Franck Mady [4], Mourad Benabdesselam [4], Gilles Mélin [5] and Mohamed Bouazaoui [1,*]

[1] Univ. Lille, CNRS, UMR 8523-PhLAM-Physique des Lasers Atomes et Molécules, F-59000 Lille, France; hicham.el-hamzaoui@univ-lille.fr (H.E.H.); bruno.capoen@univ-lille.fr (B.C.); sarah.garzandat@univ-lille.fr (S.G.)

[2] Université Jean Monnet Saint-Etienne, CNRS, Institut d'Optique Graduate School, Laboratoire Hubert Curien UMR 5516, F-42023 Saint-Etienne, France; adriana.morana@univ-st-etienne.fr (A.M.); ouerdane@univ-st-etienne.fr (Y.O.); sylvain.girard@univ-st-etienne.fr (S.G.); aziz.boukenter@univ-st-etienne.fr (A.B.)

[3] Institut Universitaire de France (IUF) Ministère de l'Enseignement Supérieur et de la Recherche, 1 Rue
Descartes, F-75005 Paris, France

[4] Université Côte d'Azur, CNRS, Institut de Physique de Nice—INPHYNI UMR 7010, F-06108 Nice Cedex 2, France; franck.mady@unice.fr (F.M.); mourad.benabdesselam@unice.fr (M.B.)

[5] Exail, Rue Paul Sabatier, F-22300 Lannion, France; gilles.melin@exail.com

* Correspondence: ismail.zghari@univ-lille.fr (I.Z.); mohamed.bouazaoui@univ-lille.fr (M.B.)

Supplementary Figure

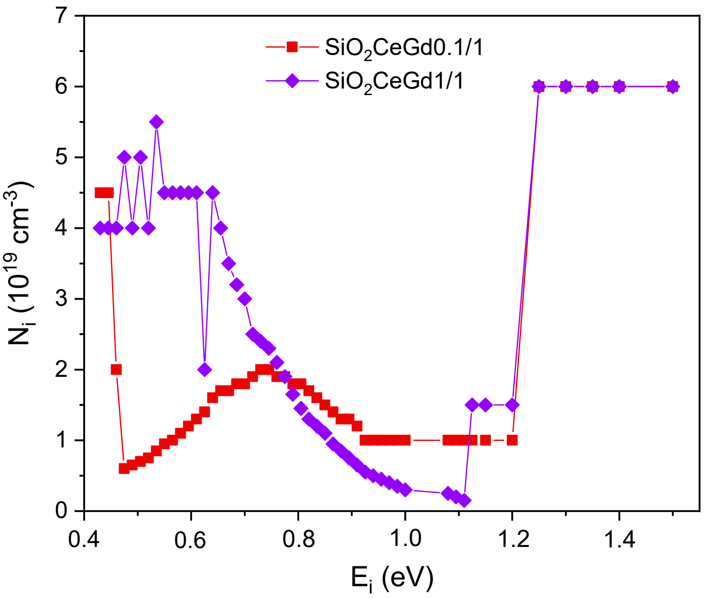

Figure S1. Concentration of trap states (N_i) taken from Table 2, versus energy.
